# Remission of Primary Hyperparathyroidism After Diagnostic Fine-Needle Aspiration Biopsies of Parathyroid Adenoma

**DOI:** 10.3390/jcm15103574

**Published:** 2026-05-07

**Authors:** Katarzyna Wojciechowska-Durczynska, Joanna Hofman, Arkadiusz Zygmunt

**Affiliations:** 1 Department of Endocrinology and Metabolic Diseases, Polish Mother’s Memorial Hospital Research Institute, 93-338 Lodz, Poland; asia.hofman9@gmail.com (J.H.); arkadiusz.zygmunt@umed.lodz.pl (A.Z.); 2 Department of Developmental Age and Adult Endocrinology, Medical University of Lodz, 93-338 Lodz, Poland

**Keywords:** primary hyperparathyroidism, fine-needle aspiration biopsy, parathyroid hormone, autoinfarction, thermal ablation

## Abstract

**Background:** The parathyroid hormone (PTH) measurements in washouts from the fine-needle aspiration biopsy (FNAB) of parathyroid adenoma could be considered in preoperative diagnostics of primary hyperparathyroidism (PHPT). Aims: Preoperative remission of PHPT following FNAB is presented, with discussion of the possible pathophysiological mechanisms and clinical implications. **Case presentation:** We describe a case of a female patient with confirmed PHPT and a suspected parathyroid adenoma who underwent FNAB with PTH washout measurement as part of the diagnostics. Following FNAB, the patient experienced normalization of biochemical parameters, accompanied by a reduction in tumor size. This outcome is presumed to be associated with autoinfarction or hemorrhage within the adenoma triggered by the biopsy procedure. **Conclusions:** This case highlights a rare but clinically significant phenomenon of FNAB-induced remission of PHPT and explains why alternative treatments such as thermal ablation may be considered to avoid surgery.

## 1. Introduction

Primary hyperparathyroidism (PHPT), characterized by hypercalcemia and elevated or inappropriately normal concentrations of parathyroid hormone (PTH), is mostly caused by a solitary parathyroid adenoma. Diagnostic approaches for PHPT include biochemical testing and imaging techniques. First-line evaluation relies on serum calcium and PTH measurements, while localization studies typically include cervical ultrasonography and technetium-99m sestamibi scintigraphy. More recently, four-dimensional computed tomography (4D-CT) has gained importance due to its high spatial and temporal resolution in preoperative localization of hyperfunctioning parathyroid tissues [1].

The fine-needle aspiration biopsy (FNAB) of suspected parathyroid adenoma may also be helpful in diagnosing PHPT [2]. The PTH measurements in washouts from the needle could be considered in the preoperative localization of the parathyroid adenoma. In a recent study by Obołończyk et al. (2022), parathyroid FNAB with PTH washout measurements demonstrated a high sensitivity of 95.6%, proving notably superior to technetium 99m sestamibi scintigraphy (52.2%) for lesion localization [3].

The article describes a case of FNAB-induced remission of PHPT. The primary aim of this study was to present this rare clinical phenomenon, while the secondary aim was to review the available literature on FNAB-induced remission and discuss its potential mechanisms and clinical implications. To the best of our knowledge, only a few cases have been published so far [2,4,5,6,7,8] (a summary of previously described cases is provided in Table 1), highlighting the rarity of iatrogenic damage leading to biochemical cure.

## 2. Materials and Methods

The data were collected from a retrospective analysis of the medical and laboratory records of a patient who was under the care of the Department of Endocrinology and Metabolic Diseases, Polish Mother’s Memorial Hospital Research Institute (Lodz, Poland). The study was carried out with prior written informed consent for publication of article and accompanying images obtained from the patient. As this was a retrospective study, the Bioethical Committee of the Polish Mother’s Memorial Hospital Research Institute declared that special ethical approval was not required (opinion No. KB113/2025).

## 3. Case Report

We report the case of a 71-year-old female referred to the Department of Endocrinology and Metabolic Diseases, Polish Mother’s Memorial Hospital Research Institute, with suspicion of PHPT. On admission, the patient complained of polydipsia and weakness. Her past medical history was remarkable for hypertension, gout, hypercholesterolemia, atrial fibrillation, osteoporosis (confirmed by dual-energy X-ray absorptiometry: lumbar spine T-score −3.7 (BMD 0.732 g/cm^2^) and total hip T-score −2.6 (BMD 0.690 g/cm^2^)) and multinodular goiter. One month before admission to the hospital, blood tests revealed a serum calcium level of 2.94 mmol/L (normal, 2.2–2.65 mmol/L) and PTH level of 338.8 pg/mL (normal, 15–65 pg/mL) (Table 1). The neck US scan displayed a nodular goiter, located behind the posterior capsule of the left thyroid lobe hypoechoic lesion, of 17 × 11 mm. The patient was qualified for FNAB of the thyroid nodules. The cytological result of FNAB indicated the II category, according to The Bethesda System for Reporting Thyroid Cytopathology (TBSRCT), in the lesion behind the posterior capsule.

**Figure 1:**
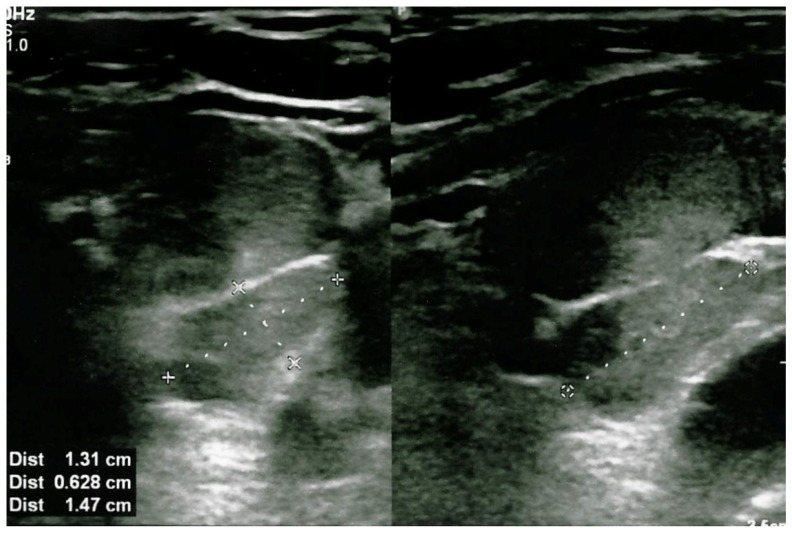
The ultrasonography revealed a well-defined, hypoechoic, homogeneous lesion measuring 13 × 6 × 15 mm, located inferior to the lower pole of the left thyroid lobe, suggestive of a parathyroid adenoma, prior to fine-needle aspiration biopsy (FNAB).

During hospitalization, PTH-dependent hypercalcemia was confirmed again: a calcium level of 3.1 mmol/L (normal, 2.2–2.65 mmol/L) and PTH level of 324.3 pg/mL (normal, 15–65 pg/mL) (Table 2). The US scan confirmed the presence of a hypoechoic lesion behind the posterior capsule of the left thyroid lobe, measuring 13 × 6 × 15 mm (Figure 1).

**Figure 2:**
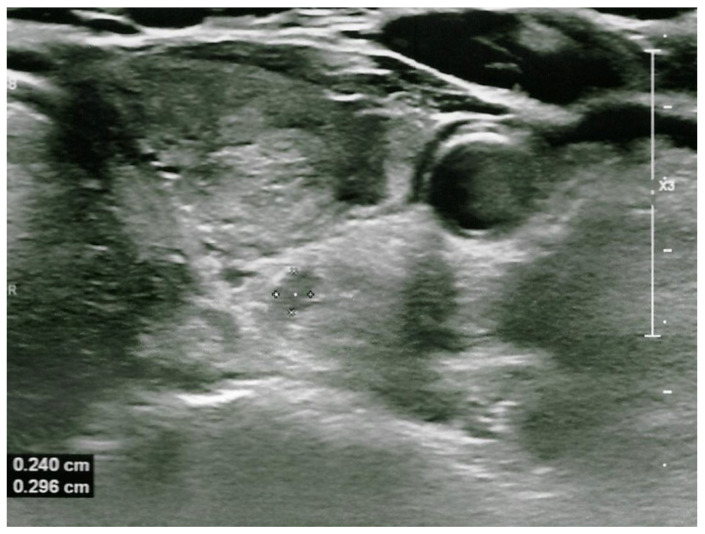
The ultrasonography demonstrated a marked reduction in the size of the previously identified parathyroid adenoma, now measuring 2.4 × 2.9 mm, following the second fine-needle aspiration biopsy (FNAB).

Regardless of the previous cytological result, the lesion was suspected of being a parathyroid adenoma, and therefore, once again, the patient underwent ultrasound-guided FNAB to collect PTH washout from the lesion. The PTH level in the washout fluid was >5000 pg/mL, confirming the parathyroid nature of the lesion. FNAB cytology was non-contributory (category I according to TBSRCT). There were no complications, and the patient did not complain of any discomfort. In order to meet the requirements before surgery, two months after the second FNAB, 99mTc sestamibi scintigraphy was performed and showed no uptake in the left posterior thyroid region. The additional laboratory testing revealed normalization of the calcium level of 2.55 mmol/L (normal, 2.2–2.65) and PTH level of 94.5 g/dL (normal, 15–65 pg/mL). The neck US showed the reduction in the parathyroid adenoma, to 4 × 4 × 5 mm (Figure 2).

After five months, biochemical parameters were still in the normal range: PTH, 77.4 pg/mL; serum calcium, 2.57 mmol/L; showing a decreasing trend. Ultrasound examination demonstrated that the parathyroid adenoma remained reduced in size. The patient did not undergo medical treatment for PHPT; she only got a recommendation for periodic follow-up.

## 4. Discussion

The mechanisms that may lead to remission of PHPT after FNAB of a parathyroid adenoma remain unclear, because most cases lack histologic confirmation, but has been suggested to be associated with autoinfarction or hemorrhage in a parathyroid adenoma [8].

The remission of PHPT may be transient or permanent, depending on the degree of cellular damage. There have been reports in the literature of PHPT remission after FNAB lasting from 12 months to 9 years [4,5,8] (Table 1). In rare cases, FNAB may result in resolution of hypercalcemia without hemorrhage or hematoma. Ing and Pelliteri (2008) [4] reported that the complete aspiration of an intrathyroidal parathyroid cyst resolved hypercalcemia for at least 16 months of post-FNAB follow-up [4].

In our case, the patient underwent two FNAB procedures in a short period of time, which could have increased the risk of bleeding or necrosis in the adenoma. FNAB affected serum calcium and PTH levels, leading to normalization. In some cases, serum calcium levels may decrease significantly after FNAB, even leading to symptomatic hypocalcemia, which requires calcium and vitamin D supplementation [9]. For this reason, close observation of patients after FNAB is advised [9].

The FNAB is not routinely recommended for suspected parathyroid nodules but may be useful in select cases; for example, when the location is atypical or a parathyroid adenoma is confused with a thyroid nodule, as in our case. Parathyroid incidentalomas detected during routine thyroid ultrasonography represent a significant diagnostic challenge, as they are often indistinguishable from thyroid nodules based on imaging features alone. In such instances, parathyroid FNAB (P-FNAB) combined with intact parathyroid hormone washout concentration (iPTH-WC) measurement is a critical tool for confirming the parathyroid origin of the lesion [10]. Markedly elevated PTH levels in the aspirate—often exceeding 5000 pg/mL, as observed in our patient, or significantly surpassing concurrent serum concentrations—provide the functional evidence required to differentiate parathyroid tissue from thyroid pathology [11].

In PHPT, imaging studies are not used for diagnosis, which remains biochemical, but for preoperative localization and surgical planning. First-line modalities include neck ultrasonography and 99mTc-sestamibi scintigraphy, while advanced techniques such as ^18F-fluorocholine PET/CT or 4D-CT are reserved for inconclusive cases [1,12]. Invasive methods, including P-FNAB with iPTH-WC, may be useful in selected patients. iPTH-WC measurement has demonstrated a sensitivity of 97.6% and a specificity of 100%, proving highly effective when scintigraphy or ultrasound results are inconclusive [3,10]. Recent research indicates that iPTH-WC values are significantly higher in adenomas with a long axis exceeding 10 mm and those possessing cystic components [13].

However, it is worth remembering that FNAB in PHPT carries potential complications, such as iatrogenic airway damage caused by bleeding, which can lead to life-threatening airway obstruction, hoarseness, or dysphagia due to pressure [14]. The inflammatory reactions and post-procedural pain have also been reported, although they are usually self-limiting. Furthermore, FNAB of parathyroid adenoma may result in histologic changes, such as fibrosis, pseudoinvasion of the capsule, and cellular atypia, which can mimic malignant parathyroid lesions, as noted by Falcetta et al. (2020) [6]. Very rarely, the procedure may result in the implantation of the parathyroid tissue, known as parathyromatosis, a potential cause of recurrent PHPT. However, a retrospective study by Balbaloglu et al. 2024 [15] found no evidence of cell seeding or parathyromatosis, suggesting that the procedure is safer than previously estimated when performed with appropriate technique [15]. Although most complications are uncommon and typically mild, their potential severity underscores the need for careful patient selection and performance of the procedure only in specialized centers with appropriate experience. Particular caution should be taken in cases of suspected parathyroid carcinoma, as FNAB is contraindicated in such situations due to the risk of tumor seeding and diagnostic difficulties [16]. This suspicion is suggested by markedly elevated levels of serum total calcium (>14–15 mg/dL) or PTH (3–10 times above the upper normal limit), as well as specific ultrasound features such as a nodule larger than 3 cm, marked hypoechogenicity, irregular margins, or local infiltration [16].

The cytologic results of FNAB of parathyroid glands have low diagnostic value because of the risk of misdiagnosis, such as mistaking a parathyroid lesion with a thyroid lesion due to overlapping cytologic features [17] or, as in our case, by accidental aspiration of thyroid cells during the passage of the needle through the thyroid gland into the parathyroid adenoma. The use of PTH immunocytochemistry serves as a valuable adjunct, substantially improving diagnostic accuracy by confirming parathyroid origin, particularly in cases with indeterminate cytomorphology, despite potential limitations related to low cellularity [18]. Another possible problem could be fibrosis, complicating surgery and definitive histologic diagnosis. Ho et al. (2021) [2] described a case of significant fibrosis in the postoperative histopathological examination of a parathyroid gland which had previously undergone FNAB, which prevented histopathological confirmation of adenoma tissue in the resected lesion.

In cases of symptomatic PTHP or when specific criteria for subclinical hyperparathyroidism are met, surgery remains the first-line treatment [19,20]; established criteria include significant hypercalcemia, reduced bone mineral density (T-score ≤ −2.5), renal impairment, or age < 50 years. In the present case, hypercalcemia and osteoporosis serve as indications for surgical intervention despite advanced age. For patients refusing surgery, management is based on a nutritional and pharmacological approach. This typically includes monitoring and the use of medications such as calcimimetics or bisphosphonates to manage calcium levels and bone density. However, there are additional circumstances in which less invasive treatments such as thermal or alcohol ablation may be considered; for example, in recurrent PHPT associated with multiple endocrine neoplasia type 1 (MEN1) or in patients with contraindications to surgery or who do not meet current criteria for surgical treatment.

The described case of our patient highlights the fragility of hyperfunctioning tissue, providing a rationale for alternative treatments such as thermal or alcohol ablation to avoid surgery [21,22]. Alternative treatment methods may be effective in achieving shrinkage of the lesion with restoration of PTH and serum calcium levels. So, for less invasive interventions to be successful, precise preoperative localization is necessary. The current clinical standard for localization has increasingly shifted toward 18F-fluorocholine (18F-FCH) PET/CT [12,23], which has achieved detection rates of 100% in some series, significantly outperforming traditional ultrasound (65.8%) and MIBI scans (65.7%). However, recent evaluations of radiofrequency ablation (RFA) suggest that while it successfully reduces lesion volume, the biochemical cure rate at one year may be as low as 30.2%, with over half of the patients (55.8%) developing normocalcemic hyperparathyroidism [10]. This indicates that RFA is a useful alternative only for those with surgical contraindications.

## 5. Conclusions

In the future, there is a need to try to understand the mechanism behind this described phenomenon of induced-FNAB remission of PHPT and assess the possibility of less invasive therapies. However, it should also be remembered that these techniques often provide only a transient reduction in calcium levels and cannot be proposed as a definitive treatment for PHPT.

## Figures and Tables

**Table 1:** Summary of previously reported cases of FNAB-induced remission of PHPT, including patient characteristics, clinical presentation, lesion type, diagnostic approach, treatment, and follow-up outcomes.

Reference	Age	Sex	Country	Symptoms	FNAB Result	FNAB Target	Outcome	Follow-Up
Ho et al., 2021 [2]	Case one: 58-year-old Case two: 37-year-old	Case one: male Case two: female	Republic of Korea	Case one: PHPT (biochemical) and osteoporosi Case two: Secondary HPT due to renal failure	Case one and two: Parathyroid adenoma	Case one and two: Parathyroid lesion	Case one and two: Remission due to hemorrhage after FNAB followed by surgery	Case one: 6 years of PHPT remission after surgery Case two: 13 years of PHPT remission after renal transplantation
Ing & Pelliteri, 2008 [4]	74-year-old	Male	USA	PHPT	Parathyroid adenoma	Parathyroid lesion	Biochemical cure after FNAB	16 months of PHPT remission
Kara et al., 2017 [5]	67-year-old	Female	Italy	PHPT	Parathyroid adenoma	Parathyroid lesion	Biochemical cure after FNAB	9 years of PHPT remission
Falcetta et al., 2020 [6]	40-year-old	Female	Italy	PHPT and renal stone disease	Parathyroid adenoma	Parathyroid lesion	Remission after FNAB	12 months of PHPT remission
Elvas et al., 2022 [7]	48-year-old	Male	Portugal	PHPT and renal stone disease	Parathyroid adenoma	Parathyroid lesion	Transient remission (forty-five days after FNAB)	Surgery
Morado da Silva et al., 2023 [8]	72-year-old	Female	Portugal	PHPT	Parathyroid adenoma, misdiagnosed as thyroid	Thyroid FNAB	Transient remission (four months after FNAB)	8 months of normocalcemia after FNAB

**Table 2:** The table summarizes the complete biochemical data, including serial measurements of serum calcium and parathyroid hormone (PTH) levels, providing an overview of their changes over time in relation to the clinical course and diagnostic procedures.

	Before First FNAB	After First FNAB	Before Second FNAB	After Second FNAB			
Date	27 June 2024	23 August 2024	9 September 2024	18 November 2024	19 December 2024	14 January 2025	11 February 2025
Inorganic phosphates [0.81–1.45 mmol/L]	1.97	0.59	0.73	-	1.06	0.88	0.87
Calcium [2.20–2.65 mmol/L]	2.96	2.94	3.10	2.55	2.70	2.54	2.57
Ionized calcium [1.2–1.32 mmol/L]		-	1.60	-	-	-	
PTH [14.9–56.9 pg/mL]	361.00	338.80	324.30	94.50	88.60	80.90	77.40
24 h urinary phosphate [12.9–42 mmol/24 h]			26.06				
24 h urinary calcium [2.5–7.5 mmol/24 h]			15.46				
Vitamin D 25-OH [deficiency < 20; sufficiency > 30 ng/mL]	24.00	23.50	29.30				
TSH [0.27–4.2 uIU/mL]			0.74				
FT3 [2–4.4 pg/mL]			2.98				
FT4 [0.93–1.7 ng/dL]			1.28				

## Data Availability

Data will be made available on request.

## Abbreviations

The following abbreviations are used in this manuscript:

PHPT	Primary Hyperparathyroidism
PTH	Parathyroid Hormone
FNAB	Fine-Needle Aspiration Biopsy
P-FNAB	Parathyroid Fine-Needle Aspiration Biopsy
iPTH-WC	Intact Parathyroid Hormone Washout Concentration
TBSRCT	The Bethesda System for Reporting Thyroid Cytopathology
US	Ultrasound
4D-CT	Four-Dimensional Computed Tomography
PET/CT	Positron Emission Tomography/Computed Tomography
18F-FCH	18F-Fluorocholine
99mTc-MIBI	Technetium-99m Sestamibi
RFA	Radiofrequency Ablation
BMD	Bone Mineral Density
TSH	Thyroid-Stimulating Hormone
FT3	Free Triiodothyronine
FT4	Free Thyroxine
